# Improvement in Survival after Paraquat Ingestion Following Introduction of a New Formulation in Sri Lanka

**DOI:** 10.1371/journal.pmed.0050049

**Published:** 2008-02-26

**Authors:** Martin F Wilks, Ravindra Fernando, P. L Ariyananda, Michael Eddleston, David J Berry, John A Tomenson, Nicholas A Buckley, Shaluka Jayamanne, David Gunnell, Andrew Dawson

**Affiliations:** 1 Syngenta Crop Protection AG, Basel, Switzerland; 2 Department of Forensic Medicine and Toxicology, University of Colombo and National Poisons Information Centre, Sri Lanka; 3 South Asian Clinical Toxicology Research Collaboration (SACTRC), Department of Clinical Medicine, University of Peradeniya, Peradeniya, Sri Lanka; 4 Faculty of Medicine, University of Ruhuna, Ruhuna, Sri Lanka; 5 Centre for Tropical Medicine, University of Oxford, Oxford, United Kingdom; 6 Syngenta, Alderley Park, Macclesfield, United Kingdom; 7 Causation Limited, Macclesfield, United Kingdom; 8 Australian National University Medical School, Canberra, Australia; 9 Polonnaruwa Base Hospital, Sri Lanka; 10 Department of Social Medicine, University of Bristol, Bristol, United Kingdom; University College London, United Kingdom

## Abstract

**Background:**

Pesticide ingestion is a common method of self-harm in the rural developing world. In an attempt to reduce the high case fatality seen with the herbicide paraquat, a novel formulation (INTEON) has been developed containing an increased emetic concentration, a purgative, and an alginate that forms a gel under the acid conditions of the stomach, potentially slowing the absorption of paraquat and giving the emetic more time to be effective. We compared the outcome of paraquat self-poisoning with the standard formulation against the new INTEON formulation following its introduction into Sri Lanka.

**Methods and Findings:**

Clinical data were prospectively collected on 586 patients with paraquat ingestion presenting to nine large hospitals across Sri Lanka with survival to 3 mo as the primary outcome. The identity of the formulation ingested after October 2004 was confirmed by assay of blood or urine samples for a marker compound present in INTEON. The proportion of known survivors increased from 76/297 with the standard formulation to 103/289 with INTEON ingestion, and estimated 3-mo survival improved from 27.1% to 36.7% (difference 9.5%; 95% confidence interval [CI] 2.0%–17.1%; *p* = 0.002, log rank test). Cox proportional hazards regression analyses showed an approximately 2-fold reduction in toxicity for INTEON compared to standard formulation. A higher proportion of patients ingesting INTEON vomited within 15 min (38% with the original formulation to 55% with INTEON, *p* < 0.001). Median survival time increased from 2.3 d (95% CI 1.2–3.4 d) with the standard formulation to 6.9 d (95% CI 3.3–10.7 d) with INTEON ingestion (*p* = 0.002, log rank test); however, in patients who did not survive there was a comparatively smaller increase in median time to death from 0.9 d (interquartile range [IQR] 0.5–3.4) to 1.5 d (IQR 0.5–5.5); *p* = 0.02.

**Conclusions:**

The survey has shown that INTEON technology significantly reduces the mortality of patients following paraquat ingestion and increases survival time, most likely by reducing absorption.

## Abstract 2

Martin Wilks and colleagues compared the outcome of paraquat self-poisoning with the standard formulation against a new formulation following its introduction into Sri Lanka.

## Editors' Summary

**Background.:**

Paraquat is a non-selective herbicide used in many countries on a variety of crops including potatoes, rice, maize, tea, cotton, and bananas. It is fast-acting, rainfast, and facilitates “no-till” farming, but it has attracted controversy because of the potential for misuse, particularly in developing countries. Better training of workers has been shown to reduce the number of accidents, and additions to the liquid formulation have contributed to a reduction in cases where paraquat was drunk by mistake—blue color and a stench agent made it less attractive to drink, and an emetic to induce vomiting aimed to reduce the time it is retained in the body.

**Why Was This Study Done?:**

Despite the changes made to the formulation, paraquat is still taken deliberately as a poison by agricultural workers in parts of the developing world. Although other pesticides cause more deaths overall, paraquat poisoning is more frequently fatal than other common pesticides. Syngenta, a commercial producer of paraquat, has developed a new paraquat formulation designed to reduce its toxicity. Syngenta introduced the new formulation in Sri Lanka, a country well known for its high level of suicides with pesticides, in 2004. This new formulation includes three components designed to reduce paraquat absorption from the stomach and intestines: a gelling agent to thicken the formulation in the acidic environment of the stomach and slow its passage into the small intestine; an increase in the amount of emetic to induce more vomiting more quickly; and a purgative to speed its exit from the small intestine, the main site of its absorption. The researchers wished to know whether the new formulation could contribute to improved survival in instances where paraquat had been ingested.

**What Did the Researchers Do and Find?:**

The researchers gathered information on the time and circumstances of when paraquat was taken, the amount that was taken, the times, and details of any vomiting, treatment, and outcomes for cases of attempted suicide by paraquat poisoning at nine large hospitals in agricultural regions of Sri Lanka from December 2003 to January 2006. In total, 774 patients were tracked in this time. Syngenta introduced the new formulation in Sri Lanka on 1 October 2004. The researchers gathered information on the formulation involved in subsequent cases, by either interview or analysis of samples. After excluding some unusual or less certain cases, they analyzed data on 586 patients, of whom 297 had deliberately taken the standard formulation and 289 the new formulation.

Although the new formulation was still toxic, the data showed an increase in the proportion of cases surviving for at least three months—from 27% (standard formulation) to 37% (new formulation), an effect that was unlikely to be due to chance. More patients vomited within 15 minutes of taking the new formulation of paraquat. Patients who died generally survived longer if they had taken the new rather than the standard formulation. The researchers estimated that the new formulation is just over half as toxic as the standard formulation, meaning that a patient was likely to suffer the same level of ill effects after taking twice as much of the new formulation compared to the standard formulation.

**What Do these Findings Mean?:**

This study was designed, funded, and led by Syngenta, the manufacturer of the standard and new formulations of paraquat but the study team included a number of independent Sri Lankan and international scientists. As the researchers observed the effects of the introduction of the new formulation across the entire country at the same time, they could not completely rule out other possible reasons for the differences in outcomes for those who had taken the two formulations, such as differences in treatment.

Despite this inherent drawback, the researchers estimate that during the study the new formulation saved about 30 lives. They conclude that the the new formulation does reduce the amount of paraquat absorbed by the body, although the study does not answer the question whether this was due to the gelling agent, the increased emetic in the new formulation or a combination of factors. The researchers suggest that the new formulation, by keeping patients alive longer, may allow doctors more time to treat patients. As no effective treatment exists at present, this benefit relies on a treatment being developed in the future.

The researchers note that the most important factor in predicting the outcome when paraquat has been taken deliberately is the dose. As a result, they suggest that the new formulation can only be one part of a wider strategy to reduce deaths by deliberate self-poisoning using paraquat. They suggest that such an integrated approach might include generic measures to reduce incidents of self-harm, reduced access to paraquat, reduced formulation strength, and improvements in treatment.

**Additional Information.:**

Please access these Web sites via the online version of this summary at http://dx.doi.org/10.1371/journal.pmed.0050049.

- The US Environmental Protection Agency has published its Reregistration Eligibility Decision for paraquat
- The Department of Health and Human Services of the US Centers for Disease Control and Prevention provides a fact sheet on how to handle paraquat and suspected cases of exposure
- The World Health Organisation has recently finished consulting on a draft Poisons Information Monograph for paraquat
- The International Programme on Chemical Safety (IPCS) has published a review of paraquat in its Environmental Health Criteria Series
- MedlinePlus provides links to information on health effects of paraquat

## Introduction

Self-poisoning with pesticides is a major public health problem in many developing countries, accounting for up to one-third of all suicides worldwide according to recent estimates [1]. While organophosphorus insecticides are by far the leading cause of morbidity and mortality in these self-poisonings, other pesticides are important in specific regions and countries [2,3]. Paraquat (1,1′-dimethyl-4,4′-bipyridinium dichloride) is a nonselective contact herbicide that has been widely used in many countries since the 1960s. Following ingestion of large amounts of concentrated formulation, the rapid development of multi-organ failure and cardiogenic shock is almost universally fatal. When smaller amounts are ingested, paraquat is actively taken up into pulmonary epithelial cells where redox cycling and free radical generation trigger a fibrotic process that may lead to death [4–7].

Survival after acute paraquat poisoning is related to the ingested amount, the circumstances of poisoning, and the formulation ingested [8]. While intentional ingestion of paraquat concentrate accounts for most recorded fatalities, the problem of unintentional ingestion prompted the introduction of formulation changes (a blue colour, a stenching agent, and an emetic) to the liquid concentrate in the late 1970s and early 1980s [9]. This change is believed to have made a major contribution to the decrease of unintentional paraquat ingestion in many countries [9,10]. However, mortality following intentional ingestion remains high, and a beneficial effect of these early formulation changes on the survival rate has not been demonstrated [11].

GRAMOXONE INTEON is a novel paraquat formulation specifically developed to decrease toxicity through a reduction in the amount of paraquat absorbed from the gastrointestinal tract following ingestion [12]. A natural alginate that immediately gels when entering the low-pH environment of the stomach has been incorporated into the formulation and the amount of emetic has been increased. These changes are designed to improve efficacy of emesis after gelling of the formulation in the stomach. An osmotic purgative, magnesium sulphate, has also been added to the INTEON formulation to help speed up the passage of remaining paraquat through the small intestine, the main site of paraquat uptake, thereby reducing overall absorption.

We carried out an observational study to compare the 3-mo survival of patients admitted to hospital following paraquat ingestion before and after the introduction of the new INTEON formulation in Sri Lanka.

## Methods

### Patients

The study was conducted in nine large hospitals (in Galle, Hambantota, Anuradhapura, Polonnaruwa, Colombo, Gampaha, Ratnapura, Kandy, and Peradeniya), covering the main agricultural areas in Sri Lanka, with the exception of the northern and eastern regions. The protocol (Text S1) was approved by four separate Ethical Committees (Text S2–S5) in Sri Lanka with responsibility for surveys/studies conducted in the nine hospitals. Patients were recruited by study physicians into the survey if they reported that they had ingested products containing paraquat or, if the pesticide ingested was unknown, the patient had clinical signs typical of paraquat poisoning (mouth lesions and/or blue colouration around the mouth). Oral informed consent to participate in the survey was sought from patients or their relatives in their native language.

### Procedures

Data on the exposure, treatment, and outcome of patients ingesting paraquat were collected prospectively from December 2003 to January 2006. Following review and approval of the registration package by the Office of the Registrar of Pesticides, the new INTEON formulation was introduced in October 2004 and stocks of the existing formulation were actively withdrawn from distributors and retailers. The pesticide, bottle, and label were similar to the standard formulation, the only differences being that the INTEON formulation was slightly more viscous, and the batch numbers differed. INTEON also included a tracer compound (500 ppm diquat) that could be detected in blood and urine following oral ingestions.

Data were collected by trained research assistants using a standardised questionnaire. Upon admission, demographic data (age, sex, and weight) were recorded together with information relating to previous treatments and transfer from a primary hospital. Details relating to the ingestion were taken: time of exposure; circumstances (intentional self-harm, accidental, homicide, or occupational); time to emesis; and number and force of vomiting episodes. The patient was asked to state the ingested volume from a range of quantities (<5 ml to >150 ml) with a variety of measuring schemes (millilitres, fluid ounces, or various-sized spoon/cup measures).

A plasma and/or urine sample was taken soon after admission, where possible. Samples were stored frozen and sent to Syngenta CTL (Alderley Park, Macclesfield, Cheshire, UK) for determination of paraquat ion concentration and detection of the tracer compound diquat ion to classify the case as either standard formulation or INTEON. Analysis was conducted using HPLC, LC-MS-MS, and LC fluorescence [13].

Details of treatments and clinical observations throughout the patients' stay in hospital and clinical outcome were recorded; if the patient was discharged from hospital, study doctors visited the patient at home at least 3 mo after the initial exposure to ascertain survival.

Cases were initially recorded on paper and then transferred to a Microsoft Access database. For quality control, a separate database was created from data collected from the medical notes by an auditor (this was not possible in two of the hospitals where permission for access to the medical records archives was refused). The two databases were compared to assess completeness of case ascertainment and to highlight differences in recording of details.

To find out whether the pattern of patient admissions to, and referrals from, hospitals not participating in the survey had changed over time, the study team contacted 147 hospitals and care units towards the end of the survey in the provinces where the study hospitals were located. Using a structured questionnaire, information was obtained from physicians who were in charge of admitting patients, or, in the case of central dispensaries, from the pharmacists.

### Case Definition and Power Calculation

Both standard and INTEON formulation cases were classified as ‘confirmed' on the basis of blood or urine analysis and as ‘probable' when bottle or label were presented (Table 1). The recording of the first two consecutive confirmed INTEON cases at each hospital was taken to indicate that INTEON use had become common in the area, and a washout period was defined for each hospital from 1 October 2004 until that time point. Cases after the washout period without sample confirmation or evidence from the bottle/label were classified as ‘possible' INTEON cases.

**Table 1:**
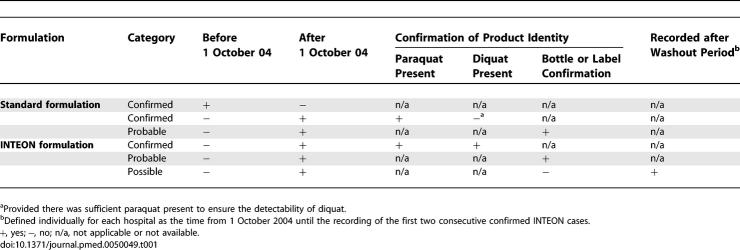
Categorisation of Cases into Standard Formulation and INTEON Formulation Groups

The power calculation was based on the Mantel-Haenszel risk ratio estimate stratified for three ingestion groups and indicated that a total of 210 cases would give > 85% power to detect a 2-fold reduction in potency for a two-sided test with significance level of 5%. It was decided to use the number of confirmed INTEON cases to close the survey in order to achieve adequate power for the sensitivity analyses. The number of confirmed cases fell below 210 after some patients were identified with admission records at more than one hospital after transferring between hospitals and other patients had to be excluded because they did not meet the study entrance criteria. However, the total number of INTEON cases (confirmed, probable, and possible) included in the analyses exceeded 210.

### Statistical Analysis

Means and proportions for baseline variables were compared between the two ingestion groups using Student's *t* test for continuous variables and the *χ* ^2^ test for categorical variables. The primary analysis compared survival among standard formulation cases before 1 October 2004 with survival among confirmed, probable, and possible INTEON formulation cases after the washout period. In sensitivity analyses, survival among all confirmed and probable standard formulation cases was compared with survival among all confirmed and probable INTEON formulation cases.

Time to death analyses were performed using both nonparametric analysis methods (Kaplan–Meier survival curve estimates and the Mantel–Cox log rank test) and semiparametric methods (Cox proportional hazards [PH] regression models). Standard errors for 3-mo survival estimates were obtained using Greenwood's method [14]. All statistical analyses were performed using Stata version 9.

Cox PH regression models were used to estimate unadjusted and adjusted hazard ratios for the INTEON formulation. Adjusted analyses always included terms for the following covariates: (a) sex, age, and weight of participant; (b) treatments received; (c) use of adsorbent; and (d) time from ingestion to presentation at a medical centre.

Estimated ingestion amount was an important factor influencing survival, but information was not available for a number of cases. Consequently, unadjusted and adjusted hazard ratios were also derived for the subset of patients who had ingestion information. Adjustment was performed with and without estimated ingestion amount in the regression model. Ingestion amount was included as a categorical variable (eight levels) but also as a continuous variable using the logarithms of the midpoint of ingestion categories. Models were also fitted to examine whether the relationship with ingestion amount differed between the two groups. Estimates of relative potency were derived using the slope of the relationship with the logarithm of ingestion amount and term for formulation group in the Cox PH model.

Variation in survival characteristics between the nine study hospitals was investigated using a gamma frailty model (proportional hazard functions with random scaling factors). In addition, evidence of nonproportional hazard functions was assessed by visual methods and by testing the significance of the interaction with the logarithm of survival time. Stratification was used to account for nonproportionality of the hazard functions.

## Results

Information was collected by the nine study hospitals on 774 patients over the study period. The numbers of participants eligible for the primary analysis and sensitivity analyses broken down by formulation are given in Table 2. The primary study population included 297 confirmed cases of standard formulation ingestion admitted before 1 October 2004 and 289 confirmed, probable and possible cases of INTEON ingestion. For sensitivity analyses all confirmed or probable cases were used (382 standard formulation and 206 INTEON cases).

**Table 2:**
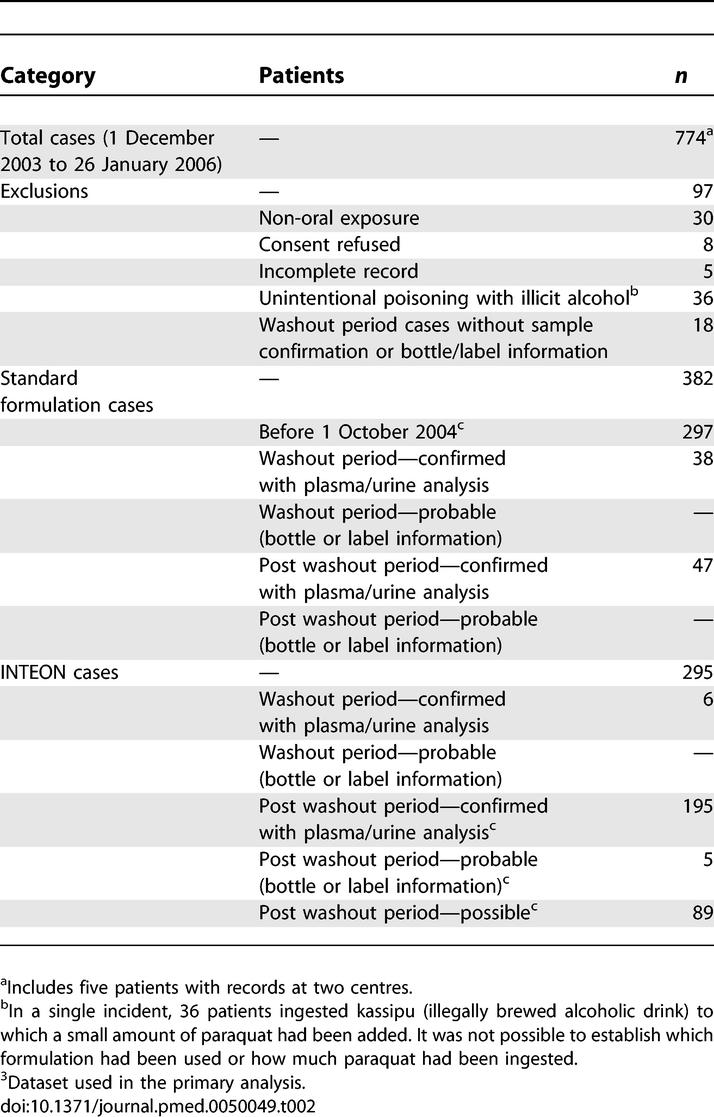
Survey Participants

The two primary study populations were similar for demographic and ingestion variables at baseline (Table 3). Most patients had ingested paraquat deliberately (93.7% of all cases). Information on ingestion volume was not available for a higher percentage of standard formulation than INTEON cases, and the distribution of cases among the ingestion subgroups was different between the two formulations.

**Table 3:**
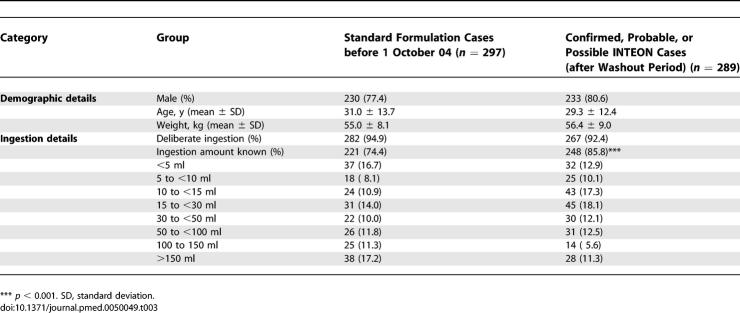
Demographic and Ingestion Details of Patients in the Formulation Groups

The clinical characteristics of the two groups were generally similar (Table 4), but a significantly higher proportion of INTEON patients vomited within 15 min of ingestion. Just over half of all patients were treated at a primary hospital before being referred to a study hospital and this proportion was higher for patients who had ingested INTEON formulation (57.8% versus 45.5%). Lavage, intravenous fluids, and prednisolone were the only treatments for which there was a significant difference between the two groups. Fewer INTEON patients received these treatments than patients who had ingested the standard formulation paraquat.

**Table 4:**
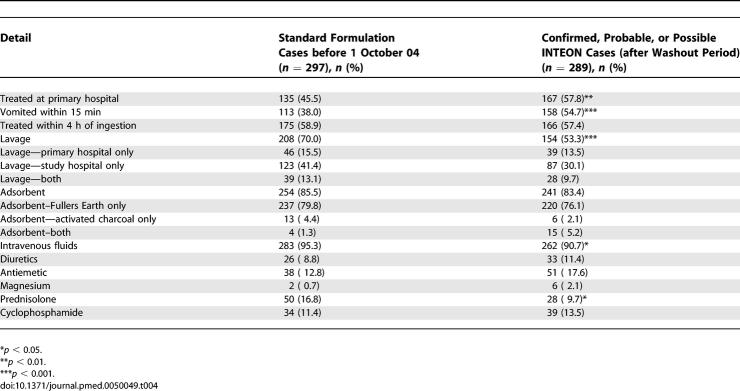
Clinical Details of Patients in the Formulation Groups

**Figure 1:**
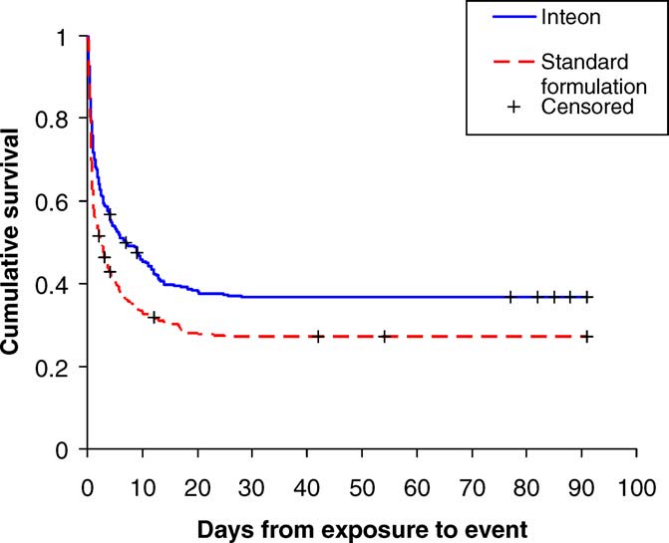
Kaplan-Meier Survival Curves for Patients Ingesting Standard and INTEON Formulation

Follow-up of patients was generally good (Table 5), but it was not possible to find out whether ten patients (4.4% of those followed up) were still alive at 3 mo. Four INTEON patients were followed up slightly early (a minimum of 11 wk after ingestion) and are described as alive in Table 5. The proportion of known survivors increased from 76 of 297 patients with the standard formulation to 103 of 289 patients with INTEON ingestion, and there was an increase in estimated 3-mo survival (Kaplan–Meier estimates) among the INTEON patients from 27.1% to 36.7% (difference 9.6%; 95% CI 2.0%–17.1%). Kaplan–Meier survival analysis (Figure 1) and log rank test indicated a significant difference between the two survival curves (*p* = 0.002). Median survival time increased from 2.3 d (95% CI 1.2–3.4 d) with the standard formulation to 6.9 d (95% CI 3.3–10.7 d) with INTEON ingestion (*p* = 0.002, log rank test).

**Table 5:**
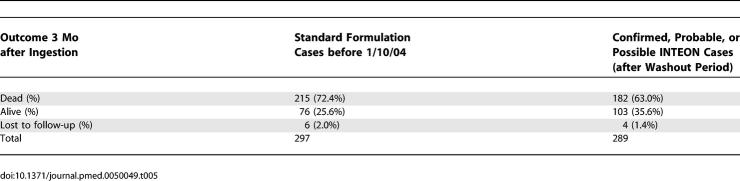
Vital Status of Patients at Three Months Following Paraquat Ingestion in the Formulation Groups

**Figure 2:**
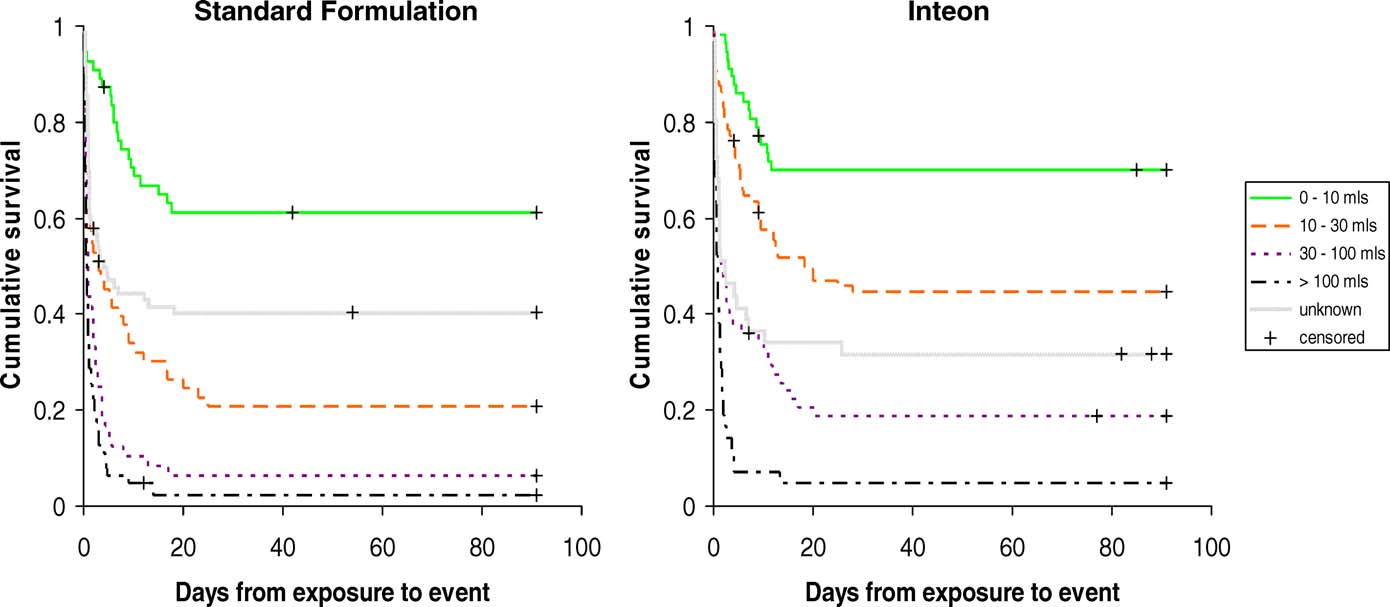
Kaplan-Meier Survival Curves by Formulation Group and Ingestion Amount

The overall improvement in survival among patients who had ingested the INTEON formulation was seen in every ingestion group except the <5 ml group, in which survival was already high. Figure 2 shows summary Kaplan–Meier survival curves for patients categorised into four ingestion groups (<10 ml, 10–30 ml, 30–100 ml, and ≥100 ml) for each formulation. In addition, survival curves are shown for patients for whom ingestion information was not available.

Survival following ingestion of INTEON was significantly better than the standard formulation (hazard ratio [HR] 0.73, 95% CI 0.60–0.89; *p* = 0.002) in an unadjusted analysis (Table 6). There was evidence of nonproportionality of the hazard functions of different hospitals, and stratification was used to account for this. However, HR changed only slightly when stratification was made for treatment centre and when covariates other than estimated ingestion amount were included in the model. Table 6 also shows that HRs were smaller when these analyses were restricted to the group of patients with ingestion information, but the fully adjusted analysis (including ingestion amount) for this latter group of patients gave an HR of 0.67 (95% CI 0.52–0.87), which is similar to that seen in the unadjusted analysis for all participants.

**Table 6:**
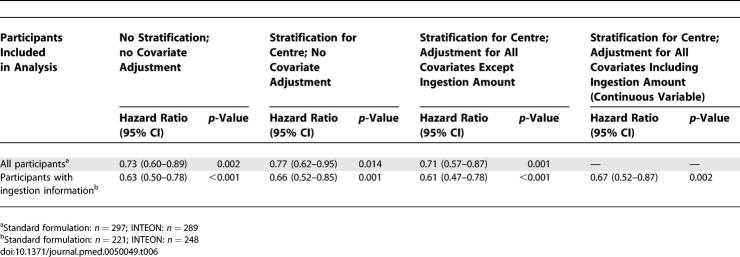
Hazard Ratios for INTEON Formulation from Cox Proportional Hazards Regression Models

Replacing the eight-level categorical variable for ingestion amount with the logarithm of the midpoint of ingestion in each category made little difference to the fit of the model (change in *χ* ^2^ = 3.62, 6 df) and there was no evidence of a different relationship with ingestion amount for the standard and INTEON formulations. The HR for a doubling of ingestion amount was 1.57 (95% CI 1.46–1.69). The strong relationship with the logarithm of ingestion amount enables an estimate to be made of the potency (toxicity) of the INTEON formulation relative to the standard formulation. Based on the subset of patients with ingestion information, the potency of INTEON was estimated to be 0.54 of the standard formulation.

Sensitivity analyses including all confirmed and probable cases gave results that were very similar to those obtained in the primary analysis. There was an increase in estimated 3-mo survival among the INTEON patients from 27.4% to 37.9% (difference 10.5%; 95% CI 2.5%–18.6%) and an HR of 0.64 (95% CI 0.50–0.82) with a potency estimate for INTEON of 0.47 of the standard formulation.

Among patients who died there was an increase in median time to death from 0.9 d (interquartile range [IQR] 0.5–3.4) for the standard formulation to 1.5 d for INTEON (IQR 0.5–5.5); *p* = 0.02. This effect was more pronounced in the sensitivity analysis, restricted to confirmed and probable cases, where the median time to death was 1.1 d (IQR 0.5–3.9) for the standard formulation but 2.5 d for INTEON (IQR 0.8–9.0); *p* = 0.001.

**Figure 3:**
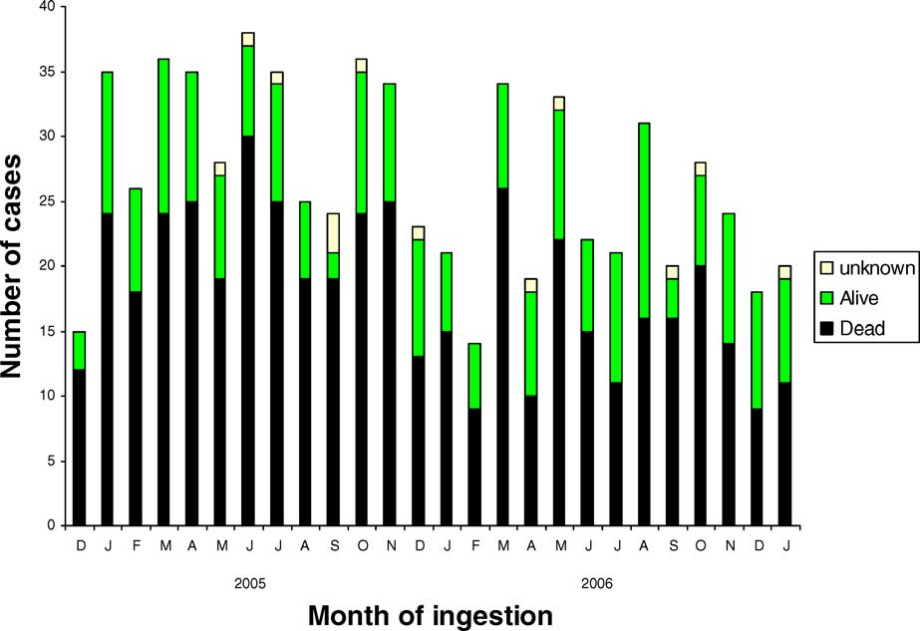
Monthly Admission Rates of Patients with Paraquat Poisoning to Study Hospitals According to Outcome at Three Months

Monthly admissions of patients with paraquat poisoning to study hospitals showed some seasonal variability, related to the use pattern of paraquat in Sri Lanka (Figure 3). However, they also suggest an overall decrease of the number of cases over time. In the separate admission and referral survey of 147 contacted hospitals and care units, 83 (56%) reported having received a total of 541 patients with paraquat poisoning. Nearly two-thirds (63%) of hospitals and care units reported no change in the number of patients seen since the introduction of INTEON, whereas 29% reported a decrease and 8% an increase. Virtually all hospitals that were able to provide information had not changed their referral pattern of paraquat-poisoned patients, and there was no difference between the larger and smaller units.

## Discussion

In Sri Lanka, pesticides are the most common means of self poisoning, with case fatality ratios more than 10-fold higher than those from self-poisoning in industrialised countries [15]. Although not the most common cause of pesticide death, paraquat has a higher case fatality ratio than other commonly ingested pesticides [16]. We have shown in this study that the development of a new formulation that turns to a gel in the stomach, slowing absorption and increasing the time available for effective emesis, increases estimated 3-mo survival from 27.1% for patients ingesting the standard formulation to 36.7% with the INTEON product. In individual terms this equates to approximately 30 lives saved within the survey due to the introduction of INTEON.

Despite much research into the mechanism of toxicity and the potential for treatment of paraquat poisoning, no specific therapy has so far been shown to affect outcome in controlled clinical studies [5,6,17]. Consequently, prevention of absorption remains an important approach to reduce paraquat toxicity. For this reason a potent emetic has been included in paraquat formulations since the late 1970s [9]. However, a beneficial effect of this measure on case fatality has not been conclusively demonstrated [11,18–22]. This may be related to the relatively large quantities of product that are often ingested in self-harm cases.

Paraquat causes mucosal damage and increases passive flux across the mucosal barrier at high concentrations [23], and peak plasma levels occur within one hour, since the liquid formulation rapidly reaches the absorptive site in the small intestine [6]. The principle of the INTEON formulation is based on the addition of alginates, which become protonated after contact with gastric acid and transformed into a gelatinous mixture. This technology is used in pharmaceuticals to treat heartburn and acid reflux [24] and to cause satiety in the treatment of obesity, by virtue of the intragastric bulking of alginates [25]. In vitro and in vivo studies have shown that the inclusion of the alginate into the formulation led to a decrease in paraquat absorption [12]. The combination of the alginate with an increased emetic concentration and magnesium sulphate added as purgative is considered to be necessary to achieve an optimum safening effect. The INTEON formulation introduced into Sri Lanka also contained a built-in surfactant system. Some of the formulation ingredients were found to gradually separate out in the bottle with prolonged storage, creating a surfactant and emetic-rich phase, and one with increased paraquat and alginate concentration. Although the formulation could be easily rehomogenised by light agitation of the bottle the overall safening effect may potentially have been suboptimal.

Although steps were taken to actively withdraw the old product from the market when the new formulation was introduced, we recognised that there would be a period in which the old product would still be with farmers. It was therefore important to unequivocally identify as many cases as possible through analysis of the marker that had been added to the INTEON product in a plasma or urine sample. However, this identification was possible only in two-thirds of the INTEON cases due to a combination of samples not being taken (e.g., in patients who were very ill on admission and died quickly) and samples with plasma paraquat concentrations so low that the diquat marker could not be detected. To reduce the number of standard formulation cases incorrectly included in the INTEON group we introduced washout periods for the centres. During the washout periods only 6/44 (14%) of patients with sample confirmation were INTEON ingestions. In contrast, 195/242 (81%) of patients with samples after the washout period had ingested INTEON. Hence, it is likely that the majority of the 89 possible INTEON cases after the washout period were correctly classified as INTEON cases. Only 18 cases with no sample information or equivocal results occurred during the washout period and had to be excluded from the survival analyses. Importantly, the sensitivity analyses excluding those patients without sample or bottle confirmation gave very similar results to the primary analysis, providing further evidence that our overall classification of cases was largely correct. The possible inclusion of a small number of standard formulation cases in the INTEON group may have had a small impact on the survival rate. However, the effect of not including possible INTEON formulation cases would have been far greater because of (a) missing cases with large ingestion volumes because of the difficulty of collecting samples from very sick patients, and (b) missing ingestions too small for the marker to be detectable in samples.

Ingestion information was not available for 26% of standard formulation cases and 14% of INTEON ingestions. The higher proportion of standard formulation cases with missing ingestion information resulted because information was not routinely collected at the start of the survey at one hospital. Many of the other patients without an ingestion amount were too ill to supply this information. Standard formulation patients with ingestion information tended to have ingested more than INTEON patients, and 29% had ingested more than 100 ml compared with 17% of INTEON patients. However, this difference in ingestion amounts would only explain a small part of the observed improvement in survival since standardising the survival rate of the standard formulation cases with ingestion information to the ingestion amount distribution of the INTEON patients only increased the estimated survival probability of standard formulation cases from 27.1% to 27.7%. Furthermore, standard formulation cases without ingestion information appeared to have ingested less than INTEON patients without ingestion information based on their higher survival rate, and the ingestion distributions of the full groups were probably closer than those of the subgroups with ingestion information.

Since the INTEON formulation was introduced in the whole country at the same time we had to rely on a before-and-after design for the survey. It is therefore possible that changes in treatment, hospital admissions, or referrals may have occurred over the period of the survey. There were some differences between the two groups in terms of treatment, with fewer INTEON patients receiving gastric lavage and prednisolone, but none of the differences were major confounders of the observed beneficial effect of INTEON on survival. Table 6 shows that the hazard ratios with and without covariate adjustment are very similar, suggesting that the differences in treatment explain very little of the group difference in survival. There is a difference in crude survival rate between those who had lavage and those who did not, but the effect disappears when adjustment is made for ingestion amount. The lower rate of lavage in the INTEON group is more likely a consequence of factors such as the higher rate of early emesis and not an explanation for improved survival. There were a number of patients who stated that they had ingested very small amounts of either formulation but had a rapid onset of emesis. It is suspected that some of these patients ingested much more than stated and hence that rapid onset of emesis in the lower exposure groups may be an indicator of misreported exposure.

The monthly admissions over the study period suggest an overall decrease of the number of cases over time. In the survey of peripheral hospitals and care units there was no indication of a change in their referral practices over time. Changes in case ascertainment and management are therefore unlikely to have substantially contributed to the improved survival noted with INTEON. However, many hospitals indicated that the number of paraquat cases had decreased. This change may relate to shifts in the general pattern of self-harm incidents, but it is also possible that fewer patients ingesting the INTEON formulation were seeking health care.

For those patients who did not survive, there was an increase in time to death for INTEON compared to the standard formulation. This difference may become important when trying to achieve improvements in the treatment of paraquat poisoning, as it may allow more time for new or existing therapies to become effective. Our data show that in Sri Lanka self-harm patients reach hospital reasonably quickly (nearly 60% are treated within 4 h), so improved treatment of poisoning cases in addition to the INTEON formulation could have a further positive effect on survival.

While our finding of improved survival of patients in the INTEON group is encouraging the data also show that the beneficial effect of the formulation is limited by the amount of product ingested, since this was the single most important predictor of survival in both groups. It is therefore apparent that formulation changes in themselves will not be sufficient to comprehensively address the problem of mortality from self-harm with paraquat. An integrated approach has recently been proposed including generic measures to reduce self-harm incidents, as well as focusing on reducing access, reducing formulation toxicity (e.g., by reducing formulation strength), and improving the treatment of poisoning [26]. However, there are clear tensions between what is desirable from public health, agricultural, and industry perspectives, and this lies at the heart of the controversy over the benefits and risks of paraquat use, in particular in developing countries. A detailed discussion of this subject is beyond the scope of this paper, but can be found elsewhere [27–29]. Nevertheless, it is evident that, as long as paraquat and other potentially harmful pesticides continue to be widely used, a comprehensive programme to prevention and management of poisoning is needed. This is why the World Health Organization (WHO) has announced a public health initiative with the overall goal to reduce morbidity and mortality from pesticide poisoning, including improved regulatory policies, epidemiological surveillance, improved medical management and mental health-care, training in the safe handling of pesticides, and community programmes that minimise the risk of intentional and unintentional poisonings [1].

In conclusion, this survey shows that the introduction of a new paraquat formulation with INTEON technology has led to a significant improvement in survival of patients with paraquat poisoning. Our statistical analyses indicate that this effect is due to a real difference between the two formulations. Patients who ingested a lethal amount of the formulation survived longer with INTEON, raising the prospect of more opportunities for treatment. These encouraging results were achieved despite suboptimal homogeneity of the formulation, and future improvements in formulation technology may reduce overall toxicity even further.

## Supporting Information

Text S1 Survey Protocol (152 KB DOC)

Text S2 Ethics Committee Approval University of Ruhuna (284 KB PDF)

Text S3 Ethics Committee Approval Anuradhapura General Hospital (322 KB PDF)

Text S4 Ethics Committee Approval General Hospital (Teaching) Kandy (284 KB PDF)

Text S5 Ethics Committee Approval National Hospital of Sri Lanka (Colombo) (627 KB PDF)

## Abbreviations

CI: confidence interval
HR: hazard ratio
IQR: interquartile range
PH: proportional hazards
