# Supplementary material for: Improvement in Survival after Paraquat Ingestion Following Introduction of a New Formulation in Sri Lanka
Source: PLoS Med. 2008 Feb 26;5(2):e49. doi: 10.1371/journal.pmed.0050049 (PMC2253611; doi:10.1371/journal.pmed.0050049)
Supplement: Text S1 — (152 KB DOC) [file pmed.0050049.sd001.doc]

# Syngenta

Alderley Park, Macclesfield,

Cheshire SK10 4TJ

**REVISED SURVEY PROTOCOL**

A survey to investigate the circumstances and survival following ingestion of paraquat in Sri Lanka.

**Sponsor: Syngenta**

**Principal Investigator: Dr M F Wilks**

**Approved by:**

**……………………………………………. ……………………………….**

Principal Investigator Date

**Agreement**

The signature of the Principal Investigator constitutes his agreement to this protocol both clinical and administrative, including the Declaration of Helsinki, and that all regulations regarding confidentiality of data will be complied with.

…………………………………………… ……………………………

Principal Investigator Date

**SYNGENTA**

Title and nature of survey: A survey to investigate the circumstances and survival following ingestion of paraquat in Sri Lanka.

Compounds to be used: None

The protocol was prepared and submitted by:

……………………………………………… …………………………

Principal Investigator Date

The above survey protocol has been considered and approved by the Syngenta Ethics Committee: approval for its implementation will be documented in the study file. The protocol has also been submitted and approved by Ethics Committees at the University of Ruhuna, Galle and by the National Hospitals in Colombo and Kandy.

**Summary of Survey Design**

Objective of study A survey to investigate the circumstances and survival following ingestion of paraquat in Sri Lanka.

Principal Investigator Dr M F Wilks.

Location of study Hospitals in Sri Lanka

Survey design Prospective epidemiological data collection using a questionnaire and hospital records. The aim is to collect baseline data, before and after the introduction of a new formulation into Sri Lanka. A blood sample will be taken for quantification of paraquat levels: urine will be collected after the introduction of the new formulation to ascertain which product the incident was associated with.

Total duration of survey Pilot survey of 3 months; main survey initially for up to a two-year period, or until data on 210 patients are available both before and after introduction of the new formulation

Type and number of patients Male and female patients with paraquat poisoning.

Primary clinical endpoints Survival of patients 3 months after ingestion

Start date June 2003

CONTENTS

Page Number

[1. SCIENTIFIC GOVERNANCE AND PERSONNEL 7](#__RefHeading___Toc87426178)

[2. Introduction 10](#__RefHeading___Toc87426179)

[2.1 Background 10](#__RefHeading___Toc87426180)

[2.2 Rationale 10](#__RefHeading___Toc87426181)

[3. Objectives 10](#__RefHeading___Toc87426182)

[4. Survey Design and methods 11](#__RefHeading___Toc87426183)

[4.1 Survey Design 11](#__RefHeading___Toc87426184)

[4.2 Data sources 11](#__RefHeading___Toc87426185)

[4.3 Subject selection 12](#__RefHeading___Toc87426186)

[4.4 Survey size and power 13](#__RefHeading___Toc87426187)

[5. Survey Compound 14](#__RefHeading___Toc87426188)

[6. Data Management and Statistical Considerations 14](#__RefHeading___Toc87426189)

[6.1 Data management and quality assurance 14](#__RefHeading___Toc87426190)

[6.2 Primary and secondary hypotheses 15](#__RefHeading___Toc87426191)

[6.2.1 Descriptive study of outcome following intentional ingestion of existing formulation 15](#__RefHeading___Toc87426192)

[6.2.2 Comparison study 15](#__RefHeading___Toc87426193)

[6.3 Statistical analysis 16](#__RefHeading___Toc87426194)

[6.3.1 Descriptive survey of outcome following intentional ingestion of existing formulation 16](#__RefHeading___Toc87426195)

[6.3.2 Comparison survey 17](#__RefHeading___Toc87426196)

[6.4 Limitations 17](#__RefHeading___Toc87426197)

[7. Administrative 19](#__RefHeading___Toc87426198)

[7.1 Survey Commencement and Discontinuation 19](#__RefHeading___Toc87426199)

[7.2 Record Retention 19](#__RefHeading___Toc87426200)

[8. Ethical Considerations 19](#__RefHeading___Toc87426201)

[8.1 Declaration of Helsinki 19](#__RefHeading___Toc87426202)

[8.2 Ethics Committee 19](#__RefHeading___Toc87426203)

[8.3 Patient Consent 19](#__RefHeading___Toc87426204)

[8.4 Confidentiality of Patients 20](#__RefHeading___Toc87426205)

[9. References 20](#__RefHeading___Toc87426206)

[10. APPENDIX 1 - Content Of Questionnaire For Qualitative Survey Of Paraquat Poisoning In Sri Lanka 21](#__RefHeading___Toc87426207)

[11. APPENDIX 2 - Survey Size And Power 23](#__RefHeading___Toc87426208)

[12. APPENDIX 3 - Odds Ratio For Comparison Study 24](#__RefHeading___Toc87426209)

[13. APPENDIX 4 - Declaration Of Helsinki 25](#__RefHeading___Toc87426210)

# SCIENTIFIC GOVERNANCE AND PERSONNEL

The scientific governance is formalised by the constituency of a Steering Group and supporting it with an independent Science Advisory Group. The composition of the two groups are as follows:

STEERING GROUP

Remit: The Steering Group is responsible for all aspects of the design and

conduct of the observational survey, specifically, its members will

ensure that the data are captured, analysed, interpreted and reported

according to established scientific and ethical principles.

Principal Investigator Dr Martin Wilks

Syngenta Crop Protection AG

Co-investigators Professor Ravindra Fernando

University of Colombo

Professor P L Ariyananda

University of Ruhuna

Dr Michael Eddleston

Oxford University

Epidemiologist Dr John Tomenson

Macclesfield

Study Co-ordinator Mr Dave Berry

Syngenta CTL

**SCIENCE ADVISORY GROUP**

Remit: The Science Advisory Group will advise the Steering Group on the

scientific aspects of survey design, conduct and interpretation.

Chairman Professor Andrew Dawson

South Asian Regional Toxicology Research

Centre, Peradeniya

Members Professor Nicholas Buckley

Australian National University

Professor David Gunnell, Dept of Social Medicine,

Bristol University

Professor Keith Hawton,

Centre for Suicide Research, University of Oxford

Dr Shaluka Jayamanne,

Polonnaruwa District Hospital

Syngenta commits to present and publish data from the survey in appropriate scientific venues and journals, and will ask the Steering Group to form the core authorship of any future publication of the survey in the scientific literature, seeking the advice and contribution of the Science Advisory Group as appropriate.

**OTHER STUDY PERSONNEL**

Study Statistician Mr I Pate

Bioanalyst Mr B H Woollen

Local Project Co-ordinator Mr J Perera (CIC )

Project Physician Co-ordinators Dr I Gunawardena (Kandy and Peradenyia)

Dr S Gunaratne (Ratnapura, Colombo and Gampaha)

Project Physician Dr L de Silva (responsible for collecting follow-up data on patients discharged from hospital).

Project Auditor Ms Nilupa Herath (reporting to Prof. A Dawson)

# Introduction

## Background

Paraquat intoxication following intentional oral ingestion continues to be a problem in some countries of the Asia Pacific Region. There have been numerous publications of individual case histories but the overall survival rates and circumstances surrounding those cases are poorly documented. The aim of this survey is to collect data on the incidence, treatment and outcome of paraquat ingestion. This will facilitate evaluation of the effectiveness of current preventative measures and treatment.

Syngenta includes an emetic, alerting agent and dye in its formulations as

part of a stewardship measure to discourage abuse of paraquat formulations.

Current methods of treatment of human paraquat poisoning are aimed

particularly at reducing the GI tract absorption of the herbicide with Fuller’s

Earth and osmotic purgatives (Smith, et. al. 1974, Syngenta, 2002). However,

there is at present no convincing clinical evidence that this treatment improves survival, and there is no specific ‘antidote’ to paraquat poisoning which is safe to use clinically.

Collection of these data will also allow the assessment of the impact

of possible future formulation changes. A new formulation, AWT (Alginate

Wall Technology) has been introduced into Sri Lanka in August 2004.

## Rationale

The data will be collected via a questionnaire, which will be completed during the periods of hospitalisation and is aimed at collecting standardised information from all hospitals involved. Plasma and urine samples will be taken. Follow up will be included to check that those released from hospital with good prognosis are not readmitted and are surviving 3 months after ingestion of paraquat.

# Objectives

The objective of this survey is to establish the survival rate following suicide attempts with paraquat in Sri Lanka. Additional data will be collected to investigate the feasibility of evaluating the effectiveness of current preventative measures and treatment. The survival rate before and after the introduction of AWT will be compared to assess the effectiveness of the new formulation.

Details of parameters which will be considered can be found in Section 4.7.

# Survey Design and methods

## Survey Design

Prospectively collected data will be used to assess whether ingestion of the new formulation of paraquat (the Alginate Wall Technology (AWT) is associated with lower case fatality than ingestion of old-formulation paraquat.

Since Jun 2003/Feb 2004 (date variance owing to the introduction of different hospitals into the survey) data on the circumstances and outcome of hospital-presenting paraquat ingestion (self-poisoning and accidental ingestion) have been prospectively recorded at up to nine Sri Lanka secondary referral hospitals (see section 6.4. This data collection has continued since the introduction of the AWT formulation in August 2004. The same survey instruments have been used to collect information on cases of intentional and accidental ingestion of paraquat after the introduction of the new formulation and to facilitate a comparison of the potencies of the two formulations.

## Data sources

Data will be collected by completion of a questionnaire (Appendix 1) for all patients reporting to hospital following intentional ingestion of paraquat. Information will be collected from the patient if possible, or relatives and friends of the patient if it is not possible to interview the patient. Details from patients who refuse to take part in the survey will not be included in any analyses.

Confirmation of paraquat ingestion will be by analysis of urine using the dithionate test or other laboratory confirmatory test for the presence of paraquat, or by clinical confirmation of evidence presented by the patient and relatives/friends including presentation of the bottle/label. A plasma sample will be taken for subsequent analysis for plasma concentration at Syngenta’s CTL in the UK.

Confirmation of AWT ingestion will be by a positive urine test for diquat bromide or by the presentation of the bottle/label. The AWT formulation will contain 0.05% diquat dibromide as a biomarker that can be detected at 0.1 ng/ml concentrations in a 3ml urine sample. Following the introduction of AWT, participating hospitals should always try to obtain a urine sample and the bottle/label. Cases of paraquat poisoning after August 2004 that are not confirmed (using urinalysis, plasma or scrutiny of the bottle/label) as having used the old formulation will be assumed to have taken AWT. A sensitivity analysis will be carried out around this assumption.

Plasma samples: a 10ml blood sample should be taken by syringe and transferred to a plastic tube containing an anti-coagulant and plasma obtained through centrifugation. The plasma sample will then be split into two aliquots; one aliquot will be put into the capped plastic vial for subsequent plasma paraquat analysis; the second aliquot will be placed in the stoppered glass vial for subsequent plasma alcohol. Note – both plastic and glass vials should only be filled to 80-90% capacity to allow for cold expansion; samples will be stored frozen and collected at intervals by CIC Ltd for analysis by Syngenta CTL.

A 10ml urine sample will be taken from all patients participating in the study; the samples will be analysed for presence or absence of the tracer to be included in Gramoxone AWT. The urine sample may be either the first void or a free-flow sample, except in the circumstances described below. Samples should be collected at least 1 hr after the exposure, to allow time for any material to be absorbed and to be excreted in the urine. It is calculated that, if present, the tracer will still be detectable in urine samples taken 24 hours plus after exposure. Samples may be voided into a plastic, glass, ceramic or metal containers and 10ml transferred into the sealed plastic tubes provided. Glass receptacles should be avoided if possible. Samples will be kept frozen at –4^o^C and collected at intervals by CIC Ltd; they will be transferred to Syngenta CTL for analysis by the Bioanalytical Group

If the patient dies after admission to the hospital but before a urine sample (for tracer detection) has been taken, then, if an autopsy is to be conducted, samples should be taken *post mortem* by the pathologist*.*. This will be dependent upon agreed consent from the relatives as well as the pathologist undertaking the examination. The consent for taking samples should be documented in the hospital records. The urine sample should be taken by bladder puncture and stored as described above in Section 1 of this amendment.

The survival of cases discharged from hospital will be ascertained at 3 months post ingestion by active follow up. A standard medical examination will be conducted at this time and any residual symptoms will be recorded. For any patients who have died between the time of discharge from hospital and follow-up the date of death will be confirmed from the death certificate.

## Subject selection

All cases of poisoning presenting to the participating hospitals - including occupational (non-oral) and accidental oral ingestion - will be included in the survey databases. No restrictions will be placed on the treatment patients receive but the treatment they receive will be documented in full. Analysis will be restricted to cases where oral ingestion has occurred and paraquat ingestion has been confirmed as described in 6.2.

**Case Definition:** cases included in the survey will be those where paraquat ingestion is reported by the patient / family report or on clinical grounds. The validity of this assessment will be tested in the sub-sample of subjects with urine / plasma samples.

**AWT Exposure definition**: cases of ingestion testing positive to diquat dibromide or where the family / patient present a bottle of the AWT formulation, will be classified as exposed to the AWT formulation or to the old formulation according to the following scheme:

| Urine or plasma analysis | Batch # on bottle | Exposure definition |
| --- | --- | --- |
| +ve Dq | Information on batch number not required | Confirmed AWT |
| -ve Dq^a^ | Information on batch number not required | Confirmed Old |
| Dq test uninformative^b^ or no sample | No bottle, AWT batch number on bottle | Probable AWT |
| Dq test uninformative^b^ or no sample | Old batch number on bottle | Probable Old |

^a^ Sufficient PQ in urine or plasma sample to detect DQ if present

^b^ Insufficient PQ in sample to test whether DQ present

All cases of poisoning that occur before 1 August 2004 will be classified as Confirmed Old cases.

## Survey size and power

Nine hospitals are participating including the University Hospital in Galle where a pilot survey was conducted between June 2003 and August 2003.

The following hospitals are participating in the survey and have been collecting information on all poisoning cases admitted since the dates shown:

Galle June – August 2003 and November 2003

Colombo January 2004

Gampaha January 2004

Kandy January 2004

Peradinya January 2004

Ratnapura January 2004

Anuradhapura* February 2004

Polonnaruwa* February 2004

Hambantoto February 2004

*Data from these 2 hospitals were being collected for a separate clinical trial from May 2002. The protocol and data collected was not identical to that required for this trial. The information required for this survey has been collected for all cases admitted to the two hospitals since February 2004.

It is expected that about 200 cases of intentional ingestion will have occurred before the AWT formulation was introduced in August 2004 and some further cases of ingestion of the existing formulation will be expected to occur after the AWT formulation is in the marketplace. The survey will have 93% power to detect a 2-fold reduction in relative potency (a reduction in overall mortality from 74% to 62%) for a series of 210 cases of intentional poisoning using the current formulation and 210 cases using the AWT formulation (Appendix 2).

# Survey Compound

Currently there are four paraquat formulations marketed in Sri Lanka, (containing 200g/l paraquat ion).

Gramoxone©

Oneshot©

Baursparaquat©

Xpress© – a small volume of this product was introduced in 2004; this product does not contain emetic.

AWT versions of Gramoxone©, Oneshot© and Baursparaquat© were introduced to Sri Lanka in August 2004. Old versions of these products were withdrawn from the distributors.

# Data Management and Statistical Considerations

## Data management and quality assurance

Data are recorded on a paper questionnaire and then transferred to the Access database by medical coordinators. Fields considered critical for analysis and data integrity are mandatory in the Access database.

An auditor, reporting to the Chair of the Scientific Advisory Panel (SAP), will audit all Access data against the paper questionnaire and the patient’s ticket. They will also go through the death records for each hospital and find each poisoning death to identify any missed patients.

All discharged patients will be followed up (at least) 3 months after leaving hospital; this assessment will be conducted by a Project Physician, reporting to the Principal Investigator and to the Chair of the SAP. For any patients who have died between the time of discharge from hospital and follow-up the date of death will be confirmed from the death certificate.

All patient records will be examined by the Principal Investigator/Project Manager and assessed for consistency of information. Any apparent anomalies will be assessed further by the Project Physician.

## Primary and secondary hypotheses

### Descriptive study of outcome following intentional ingestion of existing formulation

The primary purpose of this part of the survey is to characterise survival following ingestion of paraquat and its relationship with the self-estimated measure of the amount ingested.

Other questions of secondary interest include the following:

(i) Assessment of the relationship of plasma concentration with (a) time since ingestion and (b) amount ingested.

(ii) Assessment of whether the amount ingested (as indexed by plasma concentration) and time since ingestion are associated with the probability of survival and time to death.

(iii) Incidence of emesis following ingestion, time to emesis and influence of emesis on survival.

(iv) Does (a) the administration of absorbent (e.g. activated charcoal) and (b) delay in such administration, influence the probability of survival?

(v) Assessment of the influence of alcohol on emesis, its effect on the effectiveness of an absorbent and its relationship to the amount of concentrate ingested.

(vi) Assessment of whether amount ingested should be standardised to body weight.

### Comparison study

The primary outcome measure is 3-month case-fatality among proven cases of

paraquat poisoning. Case fatality, adjusted for the estimated amount ingested, will

be compared for cases of ingestion with the old formulation and

cases of ingestion with the AWT formulation. The principal analysis will compare all cases of Confirmed and Probable ingestion of the old formulation with Confirmed and Probable AWT cases (see 3.2).

Secondary outcome measures: It is also expected that the AWT formulation will

result in lower plasma concentrations, more intense and earlier emesis, better

function and increased survival times among those patients who do not survive to 3

months post ingestion. Plasma concentration will be treated as a secondary outcome measure, but care will need to be taken when interpreting the results because of possible bias arising from using plasma concentration results to reallocate misallocated cases to the correct formulation group.

## Statistical analysis

Statistical analysis will be undertaken by Syngenta under the guidance and supervision of the epidemiologists on the Steering Group and the Science Advisory Group.

### Descriptive survey of outcome following intentional ingestion of existing formulation

Logistic regression methods will be used to examine the relationship between 3-month survival and the following factors:

1. estimate of ingested paraquat concentrate,
2. emesis,
3. co-ingestion of alcohol,
4. plasma concentration,
5. sex and age of subject;
6. aspects of treatment received
7. absorbent use;
8. body weight;
9. time between ingestion and presentation to medical care;
10. new vs. old paraquat.

Analyses will account for clustering by treatment centre (9 hospitals). Other studies have shown that the logit of the survival probability is well described by a linear relationship with the logarithms of the plasma concentration and the time since ingestion.

Effects on survival time will be explored using both non-parametric analysis methods (Kaplan-Meier survival curve estimates and log rank trend tests) and semi-parametric methods (Cox’s proportional hazards regression model). The effects of other potential explanatory variables such as emesis (see above) will be examined by adding terms to the statistical model (Cox’s proportional hazards regression analysis).

### Comparison survey

It is assumed that the AWT formulation will behave like a reduced concentration version of the existing formulation and that the survival probably will be related to ingestion amount by a logit curve). Consequently, the odds ratio will be independent of the intake amount and provide a good summary of effect (Appendix 3). Subjects estimate the amount of paraquat ingested on an 8 point scale but in order to minimise the effect of measurement error, analyses will also be performed using ingestion collapsed into 3 categories 0-30ml, 30-150ml and >150ml.

It is expected that most mortality will occur soon after ingestion and the survival time may be subject to large error. Hence, multiple logistic regression is expected to be the principal method used to relate survival to the formulation of paraquat ingested (AWT vs. old formulation) and to estimate the potency of the AWT formulation relative to the old formulation. However, survival will be modelled using Cox’s proportional hazards regression analysis if the proportional hazards assumption is met (see below). Adjustment will be made for the effect of possible confounding factors identified in the descriptive study such as age, sex, alcohol consumption, time at start of treatment or absorbent use (for full list, see above). Effect modification by factors such as alcohol can also be examined in the regression analysis by incorporating interaction terms between the formulation and the factor of interest.

In addition, Mantel-Haenszel estimates will be used to summarise relative risk across strata and test for effect modification. In addition, nonparametric statistical tests will be performed to detect an increase in the survival probability for each amount ingested. The test can be refined by making the assumption that the survival probability decreases with intake amount for each formulation.

Time to death analyses will also be performed using both non-parametric analysis methods (Kaplan-Meier survival curve estimates and log rank trend tests) and semi-parametric methods (Cox’s proportional hazards regression models). It is possible that there may be an improvement in survival time that is not accompanied by an improvement in 3-month survival probability. The effects of other potential explanatory variables such as emesis will examined in separate stratifications in the case of non-parametric analyses or by adding terms to the statistical model (Cox’s proportional hazards regression analysis).

The new formulation is expected to result in a reduced plasma concentration curve with time, an increase in the severity of emesis and a reduced time to emesis. Appropriate parametric or non-parametric tests will be performed to test for differences between the two formulations in these parameters, adjusted for estimated ingestion amount. In addition, life table methods and the log rank test will be used to test for increases in survival time.

## Limitations

It is likely that the Probable AWT group will contain a number of old formulation cases where no bottle/label is presented and some misclassified old formulation cases (cases where the wrong bottle/label is presented). These cases will either have no test sample or samples with insufficient PQ to detect DQ. The Probable OLD group may also contain some misclassified AWT cases. Again, these cases will have no test sample or samples with insufficient PQ to detect DQ. However, the numbers of incorrectly allocated cases should diminish over time.

The effect of this misallocation can be investigated using sensitivity analyses. Cases providing urine or plasma samples that contain sufficient PQ to test for the presence of DQ will allow an estimate to be made of the proportion of old formulation cases amongst those presenting with no bottle/label over various time intervals after 1 August 2004. It will also be possible to estimate the number of cases where the wrong bottle/label is presented. In addition, it will be possible to look at the effect of excluding cases of poisoning for a range of time periods after 1 August 2004.

The use of an estimated measure of ingestion is a major limitation. However, there is no reason to expect systematic differences in the estimates of ingestion amounts between subjects who ingest the two different formulations. It is essential that the patients are not aware that there has been a change in formulation and it is important that interviewers are blind to the formulation the subject ingested.

There is good reason to expect a large degree of unintentional and intentional inaccuracy in the estimated amount ingested. However, there is still a strong difference in survival rates between the 3 ingestion groups that will be used in the analysis. Hence, it is very important to adjust for the estimated amount ingested, particularly as there is a possibility that a higher proportion of people ingesting small amounts of the AWT formulation may not seek treatment.

In order to minimise potential referral bias, it is important that the survey includes all cases of paraquat poisoning in the study areas. It is likely that some patients are sent home by primary hospitals and not referred to the secondary hospitals participating in the study. Some doctors at peripheral hospitals have said that they do not transfer since there was nothing the secondary hospital can do. The study investigators will attempt to identify all cases of poisoning in the catchment areas of the study hospitals in order to assess the degree of potential bias. If there does appear to be strong potential for bias then a separate analysis will be performed that only includes primary admissions at the participating centres.

To further assess referral bias we will monitor monthly rates of hospital-presenting paraquat self-poisoning throughout the survey period. A reduction in hospital attendance rate after the introduction of the new product is possible evidence of reduced referral from primary clinics / hospitals (although seasonal differences in the availability of paraquat and in self-harm may also contribute)

Attempts will also be made to identify patients who do not come to hospital and die at home. Work is currently underway to find the location of records of all the poisoning deaths in NC Province to find out how many paraquat poisoned patients die at home in the Anuradhapura and Polonnurawa districts. This work will be extended to other centres. The study investigators will also attempt to ascertain whether there are other cases that ingest paraquat but do not attend hospital and survive.

# Administrative

## Survey Commencement and Discontinuation

The pilot survey started in June 2003 (in Galle), followed by commencement at the other study hospitals (see Section 6.4).

An initial period of data collection is planned for two years, or until data on at least 210 patients are available both before and after introduction of the new formulation.

## Record Retention

Syngenta will retain all documentation pertaining to this survey and participating hospitals will retain individual medical records as defined by hospital policy adopted in Sri Lanka.

# Ethical Considerations

## Declaration of Helsinki

The survey will be conducted in accordance with the Declaration of Helsinki (1964) including all amendments up to and including South Africa revision (1996), see Appendix 4.

## Ethics Committee

This survey has been approved by the Syngenta CTL Ethics Committee. The protocol has also been submitted and approved by Ethics Committees at the University of Ruhuna, Galle and by the National Hospitals in Colombo and Kandy.

This revised protocol will also be submitted to these committees for information.

## Patient Consent

This study will have no influence on treatment, but patients or relatives consent will be sought at the start of the interview. Evidence of consent will be documented. Patient (or their relatives) who refuse consent will not be included in any analyses.

## Confidentiality of Patients

Syngenta will preserve the confidentiality of patients taking part in this survey. Names and information allowing the identification of patients appearing on survey documentation will not be used in reports or publications, or on any other documentation not related to this study.

# References

Smith L.L., Wright A F., Wyatt I., Rose M S. (1974). Effective treatment for paraquat poisoning in rats and its relevance to paraquat treatment in man. Br. Med. J. 4: 569-571.

Syngenta (2002). Paraquat Poisoning – A Practical Guide to Diagnosis, First Aid and Hospital Treatment.

# APPENDIX 1 - Content Of Questionnaire For Qualitative Survey Of Paraquat Poisoning In Sri Lanka

Has patient or relative consent been obtained: Yes No

Name of hospital: District /Town :

Patient Reference:

Date of admission: Time of admission:

Sex: Male or Female Weight: kg

Age: years

Was the product identified as Gramoxone Oneshot Baursparaquat

What was the reason for the exposure? Deliberate (suicide) Accidental Occupational (work-related)

When did the exposure occur? Date Time

What was the route of exposure? Oral Other - specify

What was the elapsed time to reach hospital: <1, 1-2, 2-3, 3-6, 6-9 or >9 hours

Estimate of amount of ingested: Teaspoonful (<5mls), desert spoon (5-10mls),

tablespoon or mouthful (10-15mls), two mouthfuls (15-30mls),

half small cup (30-50mls), small cup (50-100mls), large cup or

mug (100-200mls), >200mls.

Accurate estimate if known mls

Was paraquat ingestion confirmed: verbal Bottle or label Urine Test

Did the patient vomit before reaching hospital: yes or no?

If yes <15, 15-30, 31-60, 61-120, 121-240 or >241 mins following ingestion.

How significant was the vomiting slight, mild or profuse,

Did the patient only vomit once or multiple times

Has a local remedy been taken Yes No Specify

Does the patient smoke: Yes No

Was alcohol also ingested: yes No

Was a blood sample taken Yes No time, date and reference if known?

Clinical Observations on admission

Respiratory rate

Blood pressure

Pulse

APPENDIX 1 - continued

When did treatment begin - date and time?

Elapsed time to treatment:

Summary of treatment given:

Anti-emetic

Absorbent: Fullers Earth Activated charcoal Single/Multiple None

Cyclophosphamide/Prednisolone Yes No

i.v. fluids Yes No

Diuretics Yes No

Other please specify

Did the patient die in hospital? If yes date and time:

Did the doctor confirm paraquat as the cause of death Yes No

Was the patient discharged from hospital Yes No Date and time

If discharged, was the patient alive after 3 months date and time confirmed?

If not was he readmitted to hospital? Yes No

If readmitted what was the outcome? Discharged date and time

Did the patient die? Yes No

Other reason – specify

NB If the patient made a repeat suicide attempt create new form and cross reference previous form.

Name of the recorder:

Dates of the survey and follow up:

Name of the study supervisor:

# APPENDIX 2 - Survey Size And Power

An approximate calculation has been performed to calculate the power of the survey to detect a 2 fold reduction in potency. For the purpose of this calculation, three ingestion groups have been used 0-5g, 5-25g and > 25g and the following estimates of survival probabilities have been derived from the first 117 cases in the survey:

| Amount ingested | No change | 2 fold reduction in potency |
| --- | --- | --- |
| 0-30ml | 0.590 | 0.754 |
| 30-150ml | 0.180 | 0.269 |
| >150ml | 0.001 | 0.104 |

There are roughly equal numbers of subjects in the 3 ingestion groups and it has been assumed that each ingestion group will contain a third of the subjects for both formulations. Note that the rate reduction for this distribution of ingestion amounts is 0.12 i.e. overall survival will increase from 26% to 38%. It has also been assumed that the baseline dataset (ingestion of the old formulation) will include 210 cases.

Monte Carlo methods have been used to estimate the power of a test based on the Mantel Haenzsel estimate of the summary odds ratio to detect a 2-fold reduction in potency. A total of 210 cases will give 93% power to detect a 2-fold reduction in potency for a one-sided test with significance level of 5%.

Misclassification error in the subject’s estimate of the amount ingested will reduce the power of the study. It is possible to adjust the power calculation to allow for this additional source of variability in response. In addition, the new formulation might be more difficult to drink and restrict the comparison of the two formulations to a narrower range of intakes. This is another indication of efficacy, but it will reduce the power of the study to test for a change in relative potency.

The estimated survival probabilities in the lowest ingestion groups may underestimate the true survival probabilities if some of the least affected individuals are not hospitalised. This degree of underestimation will be larger for the new formulation if it does reduce the amount of paraquat absorbed. It is possible that misclassification error could result in the observed survival rate for low doses of the new formulation being lower than that of the old formulation. To minimise this bias, it might be necessary to exclude subjects from the lowest ingestion groups from some analyses. If all subjects in the lowest ingestion group (0-30ml) were excluded from the analysis, the power of the study would fall to 64%.

# APPENDIX 3 - Odds Ratio For Comparison Study

Survival will depend upon the amount of paraquat ingested. For a fixed amount of paraquat ingested, differences in survival between the two formulations can be expressed as a rate difference, a rate ratio or an odds ratio and the overall difference in survival can be expressed as a weighted combination of the individual measures of survival difference within the different ingestion groups. A measure of survival difference that is constant across the ingestion groups is preferable because the expected value of the summary measure is not dependent upon the weights and all the different groups provide estimates of the same summary measure. If the survival curve follows a logistic curve, then the odds ratio will be independent of the amount ingested and it will be appropriate to express the study hypothesis in terms of the odds ratio.

The survival curve for AWT is likely to be approximated reasonably well by the survival curve of the existing formulation with intake reduced by a constant factor i.e. equivalent to existing formulation with a reduced concentration. It does not seem likely that the difference in survival rate or the rate ratio will be the same for all ingestion groups. The dog study indicates that the new formulation leads to a significant reduction in the amount of paraquat absorbed. However, the dog study also indicates that the amount of paraquat absorbed is not reduced by a constant factor over the dose range and that the amount absorbed appears to be relatively constant over a wide range of doses before increasing at higher doses. This would imply that the survival curve in man for the AWT formulation might be relatively constant over low amounts ingested and then start to behave like that for a reduced concentration version of the standard formulation.

# APPENDIX 4 - Declaration Of Helsinki

**WORLD MEDICAL ASSOCIATION DECLARATION OF HELSINKI**

**Recommendations guiding physicians**

in biomedical research involving human subjects

Adopted by the 18th World Medical Assembly, Helsinki, Finland, June 1964, and

amended by the 29th World Medical Assembly, Tokyo, Japan, October 1975; 35th World Medical Assembly, Venice, Italy, October 1983; 41st World Medical Assembly Hong Kong, September 1989; and the 48^th^ General Assembly, Somerset West, Republic of South Africa, October 1996

**INTRODUCTION**

It is the mission of the physician to safeguard the health of the people. His or her knowledge and conscience are dedicated to the fulfilment of this mission.

The Declaration of Geneva of the World Medical Association binds the physician with the words: "The health of my patient will be my first consideration" and the International Code of Medical Ethics declares that "A physician shall act only in the patient's interest when providing medical care which might have the effect of weakening the physical and mental condition of the patient".

The purpose of biomedical research involving human subjects must be to improve diagnostic, therapeutic and prophylactic procedures and the understanding of the aetiology and pathogenesis of disease.

In current medical practice most diagnostic, therapeutic or prophylactic procedures involve hazards. This applies especially to biomedical research.

Medical progress is based on research which ultimately must rest in part on experimentation involving human subjects.

In the field of biomedical research a fundamental distinction must be recognised between medical research in which the aim is essentially diagnostic or therapeutic for a patient, and medical research, the essential object of which is purely scientific and without implying direct diagnostic or therapeutic value to the person subjected to the research.

APPENDIX 4 – continued

Special caution must be exercised in the conduct of research which may affect the environment and the welfare of animals used for research must be respected.

Because it is essential that the results of laboratory experiments be applied to human beings to further scientific knowledge and to help suffering humanity, the World Medical Association has prepared the following recommendations as a guide to every physician in biomedical research involving human subjects. They should be kept under review in the future. It must be stressed that the standards as drafted are only a guide to physicians all over the world. Physicians are not relieved from criminal, civil and ethical responsibilities under the laws of their own countries.

**I. BASIC PRINCIPLES**

1. Biomedical research involving human subjects must conform to generally accepted scientific principles and should be based on adequately performed laboratory and animal experimentation and on a thorough knowledge of the scientific literature.

1. The design and performance of each experimental procedure involving human subjects should be clearly formulated in an experimental protocol

which should be transmitted for consideration, comment and guidance to a specially appointed committee independent of the investigator and the sponsor provided that this independent committee is in conformity with the laws and regulations of the country in which the research experiment is performed.

3. Biomedical research involving human subjects should be conducted only by scientifically qualified persons and under the supervision of a clinically competent medical person. The responsibility for the human subject must always rest with a medically qualified person and never rest on the subject of the research, even though the subject has given his or her consent.

4. Biomedical research involving human subjects cannot legitimately be carried out unless the importance of the objective is in proportion to the inherent risk to the subject.

1. Every biomedical research project involving human subjects should be preceded by careful assessment of predictable risks in comparison with foreseeable benefits to the subject or to others. Concern for the interests of the subject must always prevail over the interest of science and society.

APPENDIX 4 – continued

1. The right of the research subject to safeguard his or her integrity must always be respected. Every precaution should be taken to respect the privacy of the subject and to minimise the impact of the study on the subject's physical and mental integrity and on the personality of the subject.

7. Physicians should abstain from engaging in research projects involving human subjects unless they are satisfied that the hazards involved are believed to be predictable. Physicians should cease any investigation if the hazards are found to outweigh the potential benefits.

8. In publications of the results of his or her research, the physician obliged to preserve the accuracy of the results. Reports of experimentation not in accordance with the principles laid down in this Declaration should not be accepted for publication.

9. In any research on human beings, each potential subject must be adequately informed of the aims, methods, anticipated benefits and potential hazards of the study and the discomfort it may entail. He or she should be informed that he or she is at liberty to abstain from participation in the study and that he or she is free to withdraw his or her consent to participation at any time. The physician should then obtain the subject's freely-given informed consent, preferably in writing.

10. When obtaining informed consent for the research project the physician should be particularly cautious if the subject is in a dependent relationship to him or her or may consent under duress. In that case the informed consent should be obtained by a physician who is not engaged in the investigation and who is completely independent of this official relationship.

11. In case of legal incompetence, informed consent should be obtained from the legal guardian in accordance with national legislation. Where physical or mental incapacity makes it impossible to obtain informed consent, or when the subject is a minor, permission from the responsible relative replaces that of the subject in accordance with national legislation. Whenever the minor child is in fact able to give a consent, the minor's consent must be obtained in addition to the consent of the minor's legal guardian.

12. The research protocol should always contain a statement of the ethical considerations involved and should indicate that the principles enunciated in the present Declaration are complied with.

APPENDIX 4 – continued

II. MEDICAL RESEARCH COMBINED WITH PROFESSIONAL CARE (Clinical Research)

1. In the treatment of the sick person, the physician must be free to use a new diagnostic and therapeutic measure, if in his or her judgement it offers hope of saving life, re-establishing health or alleviating suffering.

2. The potential benefits, hazards and discomfort or a new method should be weighed against the advantages of the best current diagnostic and therapeutic methods.

3. If any medical study, every patient - including those of a control group, if any - should be assured of the best proven diagnostic and therapeutic method. This does not exclude the use of inert placebo in studies where no proven diagnostic method exists.

4. The refusal of the patient to participate in a study must never interfere with the physician-patient relationship.

5. If the physician considers it essential not to obtain informed consent, the specific reasons for this proposal should be stated in the experimental protocol for transmission to the independent committee (I.2).

6. The physician can combine medical research with professional care, the objective being the acquisition of new medical knowledge, only to the extent that medical research is justified by its potential diagnostic of therapeutic value for the patient.

**III NON THERAPEUTIC BIOMEDICAL RESEARCH INVOLVING HUMAN SUBJECTS (Non-Clinical Biomedical Research)**

1. In the purely scientific application of medical research carried out on a human being, it is the duty of the physician to remain the protector of the life and health of that person of whom biomedical research is being carried out.

2. The subjects should be volunteers - either healthy persons or patients for whom the experimental design is not related to the patient's illness.

3. The investigator or the investigating team should discontinue the research if in his/her or their judgement it may, if continued, be harmful to the individual.

1. In research on man, the interest of science and society should never take precedence over considerations related to the well-being of the subject.
