# Supplementary material for: Improvement in Survival after Paraquat Ingestion Following Introduction of a New Formulation in Sri Lanka
Source: PLoS Med. 2008 Feb 26;5(2):e49. doi: 10.1371/journal.pmed.0050049 (PMC2253611; doi:10.1371/journal.pmed.0050049)
Supplement: Text S4 — (284 KB PDF) [file pmed.0050049.sd004.pdf]

11.02.2004

Dr. Indika Bandara Gawarammana.
Lecturer/Senior Registrar
Department of Medicine,
Faculty of Medicine
University of Peradeniya
Peradeniya.

Dear Sir,

Ethical clearance for the project to investigate circumstances and survival in patients with paraquat poisoning

Herewith I am sending you a copy of the letter sent me by The Chairman of Ethical Committee, General Hospital (Teaching) Kandy.

Please note to take actions mentioned by Dr.Varugunam in this letter. However Ethical clearance has been granted for your project.

Thanking you,

Director
General Hospital – Kandy.

Dr. M.G.P. SAMARASINGHE
Director
General (Teaching) Hospital
KANDY.

94 11 2472868
18/05/04 11:27 FAX 94 11 2472868 [illegible] 02
