# Supplementary material for: Improvement in Survival after Paraquat Ingestion Following Introduction of a New Formulation in Sri Lanka
Source: PLoS Med. 2008 Feb 26;5(2):e49. doi: 10.1371/journal.pmed.0050049 (PMC2253611; doi:10.1371/journal.pmed.0050049)
Supplement: Text S5 — (627 KB PDF) [file pmed.0050049.sd005.pdf]

My No.AA
The National Hospital of Sri Lanka,
Colombo.

20.04.2004

Prof.Ravindra Fernando
Senior Professor of Forensic Medicine,
Dept.of Forensic Medicine & Toxicology
Faculty of Medicine,
University of Colombo.

OBTAINING PERMISSION TO CARRY OUT A
STUDY ON PARAQUAT POISONING

Reference to your letter on the above subject, approval has been granted subject to consent from the respective consultants under whom the patients are admitted.

Director

Fc. 3.

94 11 2472868

24/05/04 12:35 FAX 94 11 2472868 [illegible] 02

My No.AA
The National Hospital of Sri Lanka
Colombo.

29.01.2004

Prof.Ravindra Fernando,
Professor of Forensic Medicine & Toxicology
University of Colombo
Dept. of Forensic Medicine & Toxicology
Faculty of Medicine,
Colombo.

REQUEST FOR A STUDY TO ESTABLISH THE CIRCUMSTANCES & SURVIVAL RATE FOLLOWING SUICIDE ATTEMPTS WITH "GRAMOXONE" IN SRI LANKA.

This refers to your letter dated 11th November,2003.

Permission for the above study has been granted subject to consent from the relevant consultants.

However the names of the consultants under whom the patients are admitted may have to be included in the study.

Director
