# Supplementary material for: Improvement in Survival after Paraquat Ingestion Following Introduction of a New Formulation in Sri Lanka
Source: PLoS Med. 2008 Feb 26;5(2):e49. doi: 10.1371/journal.pmed.0050049 (PMC2253611; doi:10.1371/journal.pmed.0050049)
Supplement: Text S2 — (284 KB PDF) [file pmed.0050049.sd002.pdf]

UNIVERSITY OF RUHUNA

ETHICS COMMITTEE

FACULTY OF MEDICINE
P.O. Box 70, Galle, Sri Lanka
Tel (09)46880
International : (+949)
Fax (09) 222314

1st September 2003

Prof. PL Ariyananda,
Department of Medicine,
Faculty of Medicine,
Galle

Dear Prof. Ariyananda,

A study to investigate the circumstances and survival following ingestion of paraquat in Sri Lanka

The Ethics committee at its meeting on 12th August 2003 reviewed your letter in response to our queries and was satisfied with your clarifications and explanations.

The committee has now granted ethical clearance for your study.

Yours sincerely,

Dr. Nelun de Silva
Chairperson
