# Supplementary material for: Improvement in Survival after Paraquat Ingestion Following Introduction of a New Formulation in Sri Lanka
Source: PLoS Med. 2008 Feb 26;5(2):e49. doi: 10.1371/journal.pmed.0050049 (PMC2253611; doi:10.1371/journal.pmed.0050049)
Supplement: Text S3 — (322 KB PDF) [file pmed.0050049.sd003.pdf]

Ethics Committee

General Hospital, Anuradhapura

Chairman:
Dr. Lakshman Gamlath

Members:

Dr. Waidyarathne
Dr. D.P.D. Wijesinghe
Dr. Ashoka Munesinghe
Dr. Anula Senevirathne
Dr. Nalika Weerakkody
Dr. E Rajasekaram
Mr. Ilangasinghe
Mrs. M.A. Premalatha
Mrs. S. Bogamuwa

Tel: General
2222261
2222262
2222263
2222166
2224810
222481
2224812
2224813

Direct:
2222266

Fax:
2225616

08.04.2004

To: Dr.Michael Eddleston

Dear Dr.Michael,

Ethical clearance for research

Title of research- Immunosuression in Paraquat Poisoning.

This is to inform you that the above research proposal has been approved by the hospital ethics committee.

Thanking you

Yours truly,

....
Chairman
Ethics Committee

Dr. L[illegible]lath,
Medical Super[illegible]dant,
General H[illegible]al,
Anuradhapura

Ethical Approval

Anurhapura (to include Polonnuwa)
Galle (to include Hambantota)
Colombo (to include Ratnapura + Gampaha)
Kandy (to include Peradeniya)
